# Cultivation and biogeochemical analyses reveal insights into methanogenesis in deep subseafloor sediment at a biogenic gas hydrate site

**DOI:** 10.1038/s41396-021-01175-7

**Published:** 2022-02-02

**Authors:** Taiki Katayama, Hideyoshi Yoshioka, Masanori Kaneko, Miki Amo, Tetsuya Fujii, Hiroshi A. Takahashi, Satoshi Yoshida, Susumu Sakata

**Affiliations:** 1 Research Institute for Geo-Resources and Environment, Geological Survey of Japan, National Institute of Advanced Industrial Science and Technology (AIST), Tsukuba, Japan; 2 Technology and Research Center, Japan Oil, Gas and Metals National Corporation, Chiba, Japan; 3 Research Institute of Earthquake and Volcano Geology, Geological Survey of Japan, AIST, Tsukuba, Japan; 4 National Institutes for Quantum Science and Technology, Chiba, Japan; 5 Present Address: Oil & Gas Upstream Business Unit, Japan Oil, Gas and Metals National Corporation, Tokyo, Japan; 6 Present Address: Institute for Environmental Sciences, Aomori, Japan

**Keywords:** Biogeochemistry, Biodiversity, Environmental microbiology

## Abstract

Gas hydrates deposited in subseafloor sediments are considered to primarily consist of biogenic methane. However, little evidence for the occurrence of living methanogens in subseafloor sediments has been provided. This study investigated viable methanogen diversity, population, physiology and potential activity in hydrate-bearing sediments (1–307 m below the seafloor) from the eastern Nankai Trough. Radiotracer experiments, the quantification of coenzyme F430 and molecular sequencing analysis indicated the occurrence of potential methanogenic activity and living methanogens in the sediments and the predominance of hydrogenotrophic methanogens followed by methylotrophic methanogens. Ten isolates and nine representative culture clones of hydrogenotrophic, methylotrophic and acetoclastic methanogens were obtained from the batch incubation of sediments and accounted for 0.5–76% of the total methanogenic sequences directly recovered from each sediment. The hydrogenotrophic methanogen isolates of *Methanocalculus* and *Methanoculleus* that dominated the sediment methanogen communities produced methane at temperatures from 4 to 55 °C, with an abrupt decline in the methane production rate at temperatures above 40 °C, which is consistent with the depth profiles of potential methanogenic activity in the Nankai Trough sediments in this and previous studies. Our results reveal the previously overlooked phylogenetic and metabolic diversity of living methanogens, including methylotrophic methanogenesis.

## Introduction

Natural gas hydrates are cage-like structures of water molecules that contain low-molecular-weight gases, such as methane, and occur in subseafloor sediments along continental margins [1]. Previous geochemical studies suggest that gas hydrates primarily consist of biogenic methane [2, 3], which indicates that methanogenesis is an essential component of the biogeochemical cycle in deep subseafloor environments.

Extensive studies have been performed to characterize the methanogenic activity and diversity in sediments at gas hydrate sites using various biogeochemical techniques. Radiotracer-based activity measurements have revealed potential methanogenesis in sediments at the Cascadia Margin [4, 5], the Nankai Trough [6, 7] and the Blake Ridge [8]. Environmental molecular sequencing has been performed to identify the microbial taxa responsible for methanogenesis. However, previous studies often failed to recover the gene sequences of methanogens from sediments at hydrate sites [9–12], possibly due to the small proportions (0.1%) of archaeal communities [13, 14]. In cultivation-based approaches, only a few methanogens have been successfully cultured from sediments at hydrate sites: novel species of *Methanoculleus* [15, 16] and *Methanococcus* [17] and enrichment cultures containing the gene sequences of methanogens [12]. Determinations of living methanogens, population, diversity, physiology and ecology, and their depth-related profiles are fundamental for understanding biogenic gas hydrate formation, but such studies are still in their infancy. In particular, the potential for methylotrophic methanogenesis remains elusive in the deep subseafloor sediments, although its occurrence and contribution to biogenic methane formation have previously been suggested in the deep terrestrial subsurface [18–20]. The concentrations of methylotrophic substrates such as methanol in sediment core samples are generally low [21], which hampers potential activity measurement using a radiotracer.

The Nankai Trough is a convergent margin with widespread bottom-simulating reflectors (BSRs); hence, a substantial amount of gas hydrates may be present [22]. In this area, the methane in the gas hydrates is of biogenic origin according to its light carbon isotopic composition (^13^C/^12^C) and the predominance of methane over ethane and propane [23]. We previously recovered the 16S rRNA and methyl-coenzyme M reductase (MCR) gene sequences assigned to methanogenic archaea from sediments on Daini Atsumi Knoll in the eastern Nankai Trough, in which up to 80% of pore spaces in sandy turbidite sediments were filled with gas hydrates [24]. In addition, polar *sn*-2-hydroxyarchaeol, which is indicative of methanogens, was detected at depths down to the BSR at this site [25].

To expand our understanding of methanogenesis in the deep biosphere, we examined methanogen diversity, population and potential activity in the sediments acquired from Daini Atsumi Knoll. Radiotracer experiments were conducted to measure the potential activities of hydrogenotrophic, acetoclastic and methylotrophic methanogenesis in the sediment slurry samples. Coenzyme F430 was quantified to estimate the living methanogen biomass. Amplicon pyrosequencing of archaeal 16S rRNA genes was used to observe the diversity of methanogens. The long-term batch incubation of the sediments with various methanogenic substrates was also performed to obtain and physiologically characterize the viable methanogens.

## Methods

### Site description and sample collection

**Fig. 1:**
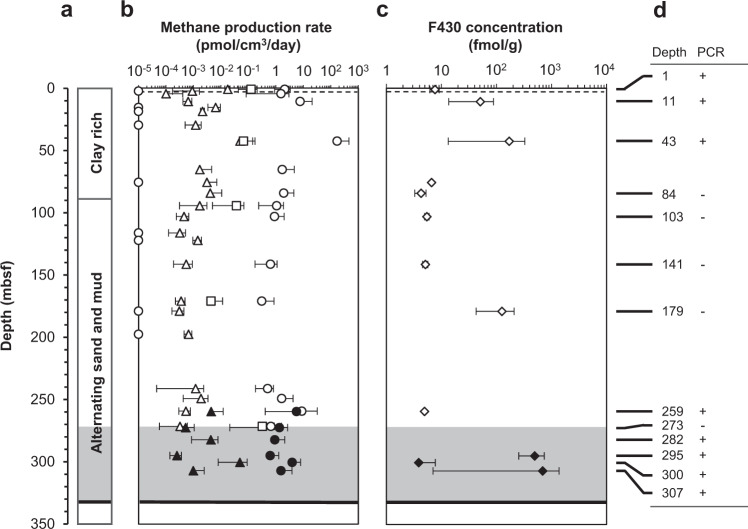
Potential for methanogenesis in the studied sites. Depth profiles of the AT1-GT1 (open symbols) and AT1-C (solid symbols) boreholes showing the sedimentary facies (**a**), methane production rates via hydrogenotrophic (circle), acetoclastic (triangle) and methylotrophic (square) pathways (**b**), F430 concentrations (**c**) and PCR amplification results of archaeal 16S rRNA genes [The presence (+) and absence (–) of PCR products] (**d**). Methane production rates below the detection limits are indicated as 10^–5^ pmol per cm^3^ per day for convenience. Error bars represent a standard deviation of triplicate (**b**) or duplicate (**c**) analysis. The SMTZ (sulfate–methane transition zone; broken line), hydrate-concentrated zone (shaded area) and BSR (bottom-simulating reflector; bold line) are indicated in the figure.

Sediment core samples were collected from two boreholes, designated AT1-GT1 and AT1-C, in Daini Atsumi Knoll (33°56’N, 137°19’E), a ridge between the forearc basin and the accretionary prism; the sediments covered the flank of the knoll. Gas hydrate-bearing strata in this area belong to the Ogasa Group, which was deposited in the middle to late Pleistocene [26]. Highly resistive anomalies in the logging data revealed an extensive gas hydrate-bearing zone distributed at 271–330 mbsf (referred to as the concentrated hydrate zone) and above the BSR (Fig. 1) [27]. The water depths of boreholes AT1-GT1 and AT1-C were 997 and 999 m below mean sea level, respectively. The distance between the two boreholes was less than 50 m. Based on the measured temperatures in the monitoring wells in the AT1 area [28], the temperatures were 3 °C at the surface of the sediments and 14 °C at the depth corresponding to the BSR (330 mbsf). The core samples were collected from shallow overburden sediments above the concentrated hydrate zone (above 273 mbsf) of borehole AT1-GT1 in February 2011 and from the concentrated hydrate zone (259–307 mbsf) of borehole AT1-C in June–July 2012. The upper layer at this site consists of clay rich slump sediment, which is considered to be a submarine landslide deposit that was transported from the top of the knoll, whereas the lower unit consists of turbidites, with alternating sand and mud sediment, originating as eroded debris that was transported by rivers and deposited in deep marine environments via turbidity currents (Fig. 1a) [26].

### Geochemical analysis

The stable carbon isotope ratios of methane were measured using a Finnigan DELTA plus XP isotope-ratio mass spectrometer (IR-MS) equipped with a Hewlett Packard 5890 gas chromatograph (GC) and a ThermoQuest combustion interface (Thermo Fisher Scientific, Waltham, MA, USA). The dissolved inorganic carbon (DIC) was measured using a DELTA V Advantage IR-MS equipped with a gas chromatography-based GasBench II system (Thermo Fisher Scientific).

The porewater was squeezed from the subcore samples using an automatic hydraulic press (AUTOFOUR/30: Carver, Inc., Summit, NJ). After the water chemistry was measured on board, the water samples were stored at −80 °C until further processing in the laboratory. The acetate concentration in the porewater sample was measured using a Prominence HPLC system (Shimadzu Co. Ltd., Kyoto, Japan) equipped with an electrical conductivity detector. The concentrations of methylated compounds, methanol, dimethyl sulfide and trimethylamine (TMA) in the porewater sample were measured using GC/MS (Agilent 5973, Agilent Technologies, Santa Clara, CA, USA) equipped with a headspace sampler (Agilent 7697A, Agilent Technologies).

### Methanogenic activity measurement using a radiotracer

The methane production rates were measured using radiotracer experiments as previously described [5, 6]. Samples were taken from the centre of the sediment core and mixed at a 1:2 volume ratio with artificial sulfate-free seawater containing 28.4 g l^−1^ NaCl, 5.7 g l^−1^ MgCl_2_·6H_2_O, 0.7 g l^−1^ KCl, 0.09 g l^−1^ KBr and 1 μg l^−1^ resazurin in an anaerobic chamber [24]. Prior to mixing, the artificial seawater was purged with oxygen-free N_2_/CO_2_ gas (80:20, v/v) and autoclaved. Forty millilitres of the slurry sample was dispensed in a 100-ml serum vial sealed with a butyl rubber stopper and aluminium crimp. The radiotracer ^14^C-bicarbonate (10 μl, 199 kBq), [2-^14^C]-acetate (10 μl, 42 kBq) or ^14^C-methanol (10 μl, 114 kBq) was then injected into the vial. The vials were incubated at 9 °C to approximate the in situ temperature under an atmosphere of N_2_/CO_2_ (80:20, v/v) without shaking for 14, 28 and 42 days.

After incubation, the reactions were terminated with the addition of 1 M NaOH. The produced ^14^CH_4_ was oxidized to ^14^CO_2_ by flushing the bottle headspace with He through a furnace containing CuO. ^14^CO_2_ was collected in vials containing 2-phenethylamine and mixed with a 10-ml Permafluor E^+^ scintillation cocktail (Perkin Elmer, Waltham, MA, USA). The total ^14^C activities were determined in triplicate for each incubation period with a Tri-Carb 3100TR liquid scintillation counter (Perkin Elmer). The ^14^C activity at zero time was used as a control. The methane production rate was calculated using the equation *a*_*p*_ / (*a*_*r*_ · *t*) · ψ · *C*, where *a*_*p*_ and *a*_*r*_ are the activities of the product and the added reactant (i.e., CO_2_, acetate or methanol), respectively; *t* is the incubation period; ψ is the porosity of the sediments; and *C* is the in situ concentration of the reactant. The alkalinity in the porewater sample was previously measured [24].

### Extraction and quantification of coenzyme F430

The coenzyme F430 in sediment samples was extracted and quantified with the modified methods of Kaneko et al. [29]. In method 1, the sediment core samples that were stored at <−25 °C were extracted with 1% formic acid (pH 2) by ultrasonication on ice, followed by centrifugation to recover the supernatant. This step was repeated three times. The supernatant was introduced to an anion exchange column (Q Sepharose column; GE Healthcare, Tokyo, Japan) that had been equilibrated with 50 mM Tris/HCl (pH 7.5) and washed with deionized water prior to use. The recovered eluent was introduced to a C_18_ SPE column (Sep-Pack; Waters Corp. Milford, MA, USA) that had been washed with methanol and conditioned with 1% formic acid. The F430 fraction on the column was eluted with 100% methanol. The F430 fraction was dried and reacted with BF_3_/methanol to convert F430 to its pentamethyl ester (F430M). In method 2, all treatments before methyl esterification were conducted without glassware, and liquid–liquid extraction with H_2_O and dichloromethane was conducted before methyl esterification to remove lipids. The F430M obtained by both methods was quantified using an LC-MS/MS (Agilent 6490 triple quadpole LC-MS coupled with an Agilent 1260 LC system). The concentration of F430 was calculated by an external standard method using the concentration of the known coenzyme F430 standard, which had been prepared using methanogenic granules as previously described [30].

### 454-Pyrosequencing of archaeal 16S rRNA genes from sediment core samples

The DNA extracted from the sediment core samples in this study for the archaeal 16S rRNA gene analyses was the same as that in Katayama et al. [24] for the prokaryotic 16S rRNA and *mcrA* gene analysis.

The V3 and V4 regions of the archaeal 16S rRNA genes were amplified with AmpliTaq Gold 360 DNA polymerase (Thermo Fisher Scientific) using the Arc806R primer fused with 454-specific adaptor A and 6-bp barcode sequences and the Arc109F primer fused with adaptor B. The primers used in this study are listed in Supplementary Table S1. No amplicons were obtained from the 84, 103, 141, 179 and 273 mbsf samples. The cycling conditions were 95 °C for 10 min, followed by 35–40 cycles of 95 °C for 30 s, 50 °C for 30 s and 72 °C for 60 s, and a final extension period of 7 min at 72 °C. Under this condition, the PCR product from the no-template control was not observed in agarose gel electrophoresis. Six replicates of the PCR products for each sample were pooled and purified using a MonoFas DNA purification kit (GL Sciences, Tokyo, Japan). Pyrosequencing was performed using a 454 Life Sciences GS FLX Titanium platform (Roche, Basel, Switzerland) at Hokkaido System Science Co., Ltd. (Sapporo, Japan).

### Enrichment culture and isolation of methanogens

The sediment slurry (prepared as described above) was dispensed in 40-ml aliquots into a 100-ml serum vial sealed with a butyl rubber stopper and an aluminium crimp under an atmosphere of N_2_/CO_2_ (80:20, v/v). The slurry sample was supplemented with either H_2_/CO_2_ (80:20; 0.1 MPa) plus 10 mM acetate or 10 mM methanol plus 10 mM TMA. To prevent the growth of bacteria that compete with methanogens (i.e., acetogenic bacteria), a slurry sample supplemented with the respective methanogenic substrates and bacteria-specific antibiotics, i.e., kanamycin and vancomycin (50 µg ml^−1^ each), was also prepared. The vials were incubated at 9 °C without shaking. The sediment slurry sample from 43 mbsf was also cultured without methanogenic substrates at 9 and 25 °C. The time courses of methane production and hydrogen consumption were measured via GC with a thermal conductivity detector.

The methane-producing cultures were subsequently transferred to a sulfate-free saline mineral medium (WS medium) [31] supplemented with either H_2_/CO_2_ (80:20; 0.1 MPa) plus 2 mM acetate, 20 mM acetate or 10 mM methanol plus 10 mM TMA. Titanium(III) citrate (0.2 mM) and 2-mercaptoethane sulfonic acid (5 mM) were also added to the medium. Cultivation was performed at 25 °C under an atmosphere of N_2_/CO_2_. After the methane production was terminated, 5 ml of the methane-producing cultures, including initial enrichment cultures as described above, were harvested and used for the clone library analysis as described below.

A deep agar slant method combined with a dilution-to-extinction method [31] was used to isolate the methanogens. The antibiotics, kanamycin and vancomycin, were added to the media at concentrations of 50 μg ml^−1^ each. The purity of the isolate was verified by microscopic observation and 16S rRNA gene amplification and sequencing.

Strains 1H1Hc7, 25XMc2 and MSS35 were deposited in the Japan Collection of Microorganisms (JCM) under JCM numbers JCM 39199, JCM 39200 and JCM 39201, respectively.

### Sanger sequencing from methanogenic enrichment cultures and isolates

Total DNA was extracted from the enrichment cultures and isolates using an ISOIL and ISOPLANT II DNA extraction kit (NIPPON GENE, Tokyo, Japan), respectively, and according to the manufacturer’s protocol. The archaeal and bacterial 16S rRNA genes were amplified from enrichment cultures using the primer pairs Arc21F and Univ1490R and Bac8F and Bac1492R, respectively. The products were purified using a MonoFas DNA purification kit, cloned into the pCR4-TOPO vector (Thermo Fisher Scientific) and sequenced using the dideoxynucleotide chain termination method with BigDye terminator reagents (Thermo Fisher Scientific).

The 16S rRNA and *mcrA* gene sequences of methanogenic isolates were determined. The ME3MFe and ME2r’ primer pair was used for *mcrA* gene amplification. The bacterial 16S rRNA gene was amplified using the primer pair Bac8F and Bac1492R to check the purity of the methanogenic isolate.

### Sequence analysis

The 454-pyrosequencing reads were analyzed using Mothur ver. 44 [32] as previously described [20] with the following modifications. Each unique operational taxonomic unit (OTU) was classified by a Bayesian classifier and on the Silva taxonomy SSU Ref NR99 release 138 datasets [33] with a confidence threshold of 80%. The taxonomic information of the OTUs (>99% sequence similarity) was obtained from the majority consensus taxonomy of the sequences within the OTU with a consensus confidence threshold of 80%. The known methanogens were retrieved from this majority consensus taxonomy for the OTUs of the 16S rRNA gene sequences. OTU clustering (>99%) was also performed using pyrosequencing reads and sequences of methanogenic culture clones and isolates to examine whether the sequences of culturable methanogens were also detected in the original sediment core samples.

The representative 16S rRNA gene sequences of methanogenic culture clones and isolates were aligned with their relatives. Neighbour-joining and maximum-likelihood trees were constructed using MEGA v.6 [34] and TREEFINDER [35], respectively.

The GenBank/EMBL/DDBJ accession numbers of the 16S rRNA gene sequences were LC183829 to LC183864. The 454 sequences were deposited in the DDBJ Sequence Read Archive database under accession number DRA005478.

### Cultivation of methanogenic isolates at different temperatures

Strains 1H1Hc7 and 25XMc2 were cultured using WS medium amended with 20 mM formate, 4 mM acetate, 0.2 mM titanium III citrate and 5 mM 2-mercaptoethane sulfonic acid at 4, 9, 20, 25, 30, 40, 45, 55 and 65 °C. The time course of methane production was measured in triplicate.

## Results

### Geochemistry of gas and porewater

The stable carbon isotopic ratios of methane (δ^13^C-CH_4_) in the studied sediments ranged from −82.4 to −61.1‰ (Supplementary Table S2), suggesting their origin to be biogenic [36]. In addition, they were lower than those of DIC (δ^13^C-DIC) by an average of −73‰ (Supplementary Table S2), a value consistent with the carbon isotope fractionation associated with hydrogenotrophic methanogenesis [36]. We, therefore, conclude that the methane in the sediments was biologically produced primarily via the carbonate reduction pathway.

The methanol concentrations in porewater samples were successfully determined, ranging from 6.7 to 75.6 μmol (Supplementary Table S3), and permitted the estimation of methylotrophic methanogenic activity rates at 1, 43, 94, 171 and 271 mbsf, as described below. The methanol concentrations were comparable with those in shallow sediments from the Japan Sea (shallower than 30 mbsf) [21]. Dimethyl sulfide and TMA, which can also be used in general by methylotrophic methanogens, were below the detection limits (<20 nM).

### Potential methanogenic activity rates

The ^14^C tracer experiments detected the potential activity of hydrogenotrophic methanogenesis in most sediments (21 out of 30) from the near-surface to the BSR (Fig. 1b), with activity rates ranging from 0.31 to 170 pmol cm^−3^ d^−1^. All nine sediment samples with activity rates below the detection limit were situated shallower than 200 mbsf. The potential methanogenic rates were comparable with those observed on Cascadia margin [4, 5]. The potential activities of methylotrophic and acetoclastic methanogenesis were also detected, ranging up to 0.12 and 0.049 pmol cm^−3^ d^−1^, respectively. Relatively high rates observed via the hydrogenotrophic pathway are consistent with the carbon isotope fractionation between methane and DIC, as well as the methanogenic community composition (see below).

### Distribution of coenzyme F430

F430 is a key prosthetic group of MCR that catalyses the last step of methanogenesis and the first step of methane oxidation [37]. Therefore, it is generally derived from both methanogens and anaerobic methanotrophic archaea (ANME) [38, 39]. A relevant point to be noted is that methanogens can only produce methane, while ANME may perform not only methane oxidation but also methane production [40, 41]. In this study, however, F430 detected in the sediment might be derived mostly from methanogens because the *mcrA* gene sequences obtained from the same DNA samples indicated the predominance of methanogens over ANMEs [24]. Due to the unstable nature of F430, the compound would not remain intact in the environment [38, 39]. We, therefore, consider that most of the F430 detected in this study is a living biomarker of methanogens. In all 13 sediment samples analyzed F430 was successfully detected with a concentration ranging from 3.9 to 689 fmol per g sediments (Fig. 1c). The predominant peaks of F430 concentration were observed in the 43, 179, 295 and 307 mbsf samples. This depth profile was roughly consistent with those of potential methanogenic activity rates (Fig. 1b) and molecular analysis of archaeal 16S rRNA genes (Fig. 1d): low levels of methane production rates and F430 and no amplification of archaeal 16S rRNA genes were observed within the upper part of turbidites with alternating sand and mud sediment (approximately 80–250 mbsf). Based on the calculation by Kaneko et al. [29], the measured F430 concentrations were equivalent to approximately 10^6^−10^8^ cells of methanogens per g sediments. These levels of F430 contents were comparable with those at another site of the eastern Nankai Trough and off the Shimokita Peninsula and one or two orders of magnitude lower than those in paddy soils [29].

### Archaeal and methanogenic community compositions in sediment core samples

**Fig. 2:**
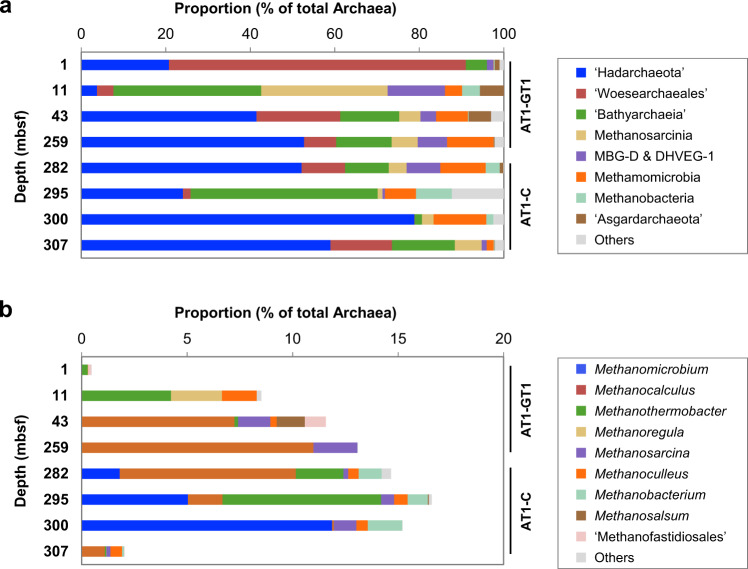
Archaeal community structures in the studied sites. Community compositions of archaea (**a**) and methanogens (**b**) in the sediment core samples based on the archaeal 16S rRNA gene sequences. Taxonomic groups that accounted for >5% and >1% are indicated in (**a**) and (**b**), respectively. DHVEG Deep sea Hydrothermal Vent Euryarchaeotal Group, MBG-D Marine Benthic Group D.

To evaluate the methanogen diversity in sediment core samples, 454-pyrosequencing analysis of archaeal 16S rRNA genes was performed because in our previous study [24], prokaryotic 16S rRNA gene sequence analysis showed that methanogens constituted a major proportion in total archaea but not in total prokaryotes. After quality filtering, pyrosequencing yielded 6857–13,076 reads per sample. At a cut-off value of 97% similarity, a total of 2230 OTUs were obtained. The major taxonomic groups (>5% of the total reads in at least one sample) were ‘Hadesarchaeota’, ‘Woesearchaeales’, ‘Bathyarchaeia’, *Methanosarcinia*, Marine Benthic Group D, *Methanomicrobia*, *Methanobacteria* and ‘Asgardarchaeota’ (Fig. 2a). The ‘Asgardarchaeota’ sequences were primarily detected in shallower sediment samples (above 43 mbsf). Intriguingly, 36–99% of the sequences in the class *Methanosarcinia* were further assigned to ‘Syntrophoarchaeaceae’, which contains a butane-oxidizing archaeon *Ca*. ‘Syntrophoarchaeum’ [42], although nonmethane gaseous hydrocarbons, including butane, were almost absent in the study area [23].

A total of 456 OTUs of the 16S rRNA gene sequences belonging to known methanogens were detected in all of the examined sediment core samples and accounted for 0.5–16.6% of the total archaeal sequences (Fig. 2b). The lowest proportion of methanogenic sequences was found at 1 mbsf and was located above the sulfate–methane transition zone [24]. The sequences of hydrogenotrophic *Methanocalculus*, *Methanoculleus* and *Methanobacterium* were also detected in clone libraries of the methyl-coenzyme M reductase (*mcrA*) genes [24], which were previously analyzed from the same DNA samples used for the analysis in this study. Consistent with this previous *mcrA* gene analysis [24] and the present radiotracer activity measurements, hydrogenotrophic methanogens dominated throughout the depths (62–97% of the total methanogenic sequences in a sediment sample), followed by methylotrophic methanogens (0–27%) and acetoclastic methanogens (0–14%) (provided that all of the sequences of *Methanosarcina* are classified into both methylotrophic and acetoclastic groups, and all sequences of *Methanofastidiosales* are classified into both hydrogenotrophic and methylotrophic groups). Among the methanogenic genera found in this study, only *Methanoculleus* and *Methanosarcina* were previously recovered from sediments at hydrate sites in the 16S rRNA gene sequencing analysis [43–45].

### Enrichment cultures of methanogens

Batch-type cultivation was used to obtain viable methanogens from the sediment core samples. After the incubation of 30 sediment slurry samples for up to approximately 2 years, 55% (v/v) of the maximum theoretical yield of methane was produced from seven samples supplemented with methylated compounds (i.e., methanol and TMA), whereas methane with an average of <0.1% of the maximum theoretical yield was produced from eight cultures supplemented with H_2_/CO_2_ and acetate (Supplementary Table S4). In hydrogen- and acetate-amended cultures, the acetate concentration increased together with the depletion of hydrogen after cultivation, suggesting the occurrence of the acetogenic activity. To prevent acetogen growth, the incubation of sediment slurry supplemented with H_2_/CO_2_, acetate and bacteria-specific antibiotics was also performed. However, none of these cultures showed methane production. In contrast, methane production was observed in five culture samples supplemented with methylated compounds and antibiotics (Supplementary Table S4).

### Phylogenetic diversity of culturable methanogens

**Fig. 3:**
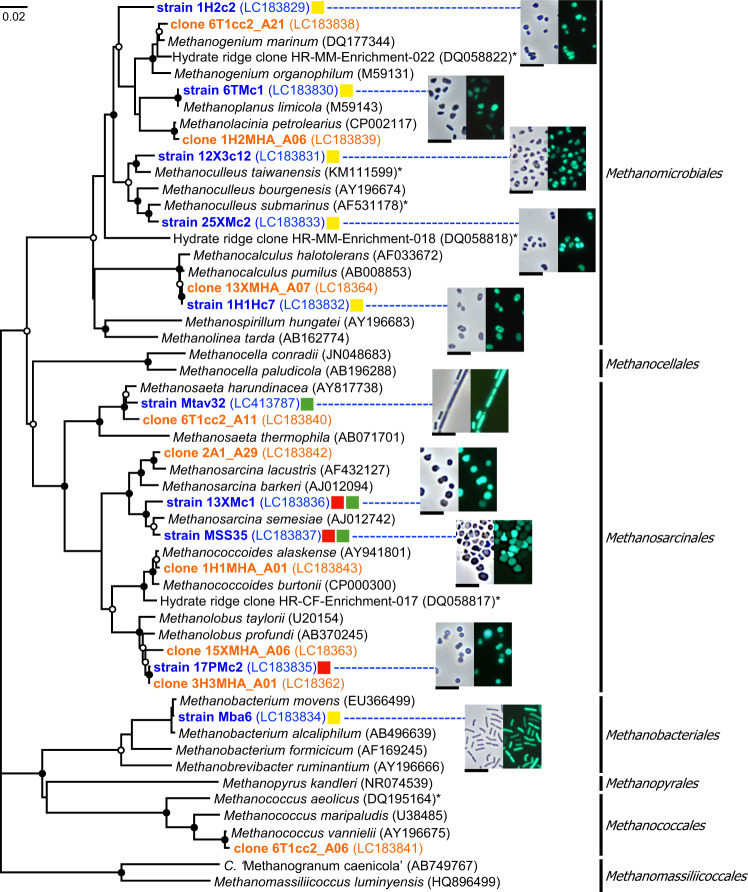
Neighbour-joining tree showing the diversity of methanogenic isolates (blue) and representative clones in enrichment cultures (orange) based on the 16S rRNA gene sequences and phase-contrast (left) and fluorescence (right) micrographs of the cell morphologies of the isolates. The asterisks indicate the species or clones that were obtained from hydrate-bearing subseafloor sediments in previous studies. The substrate utilization of the isolates is indicated by coloured squares: H_2_/CO_2_ (yellow), methanol (red) and acetate (green). Filled and open circles indicate branches with bootstrap values >80% and >50%, respectively, in both neighbour-joining and maximum-likelihood trees. Bar: 0.02 nucleotide substitutions per position. In micrographs, the bars represent 5 μm.

A total of ten methanogenic isolates were obtained together with nine enrichment culture clones assigned to methanogens (Fig. 3 and Supplementary Table S5). The isolates showed methane production from H_2_/CO_2_, acetate and/or methylated compounds, and the cells showed F_420_-autofluorescence (Fig. 3). These methanogenic cultures were obtained from various sediment depths. Methylotrophic methanogens were most frequently recovered in terms of sediment sample numbers. Acetoclastic and methylotrophic methanogens, such as *Methanosarcina*, *Methanolobus* and *Methanosaeta*, were not previously isolated from sediments in hydrate areas, although an enrichment culture containing methylotrophic *Methanococcoides* was previously obtained [12] (Fig. 3). Based on the 16S rRNA gene sequences, the isolates and culture clones were classified into the known methanogenic orders *Methanomicrobiales*, *Methanosarcinales*, *Methanobacteriales* and *Methanococcales*. The 16S rRNA gene sequences identical or closely related (>99% sequence similarity) to the isolates *Methanocalculus* sp. 1H1Hc7, *Methanoculleus* sp. 25XMc2 and *Methanosarcina* sp. MSS35 were found in 454-pyrosequencing data retrieved directly from the sediments and accounted for 0.08–76.4% and 0.01–11.2% of the total methanogenic and archaeal sequences, respectively, in each sediment sample (Supplementary Table S5). The *mcrA* gene sequences identical to *Methanocalculus* sp. 1H1Hc7 and *Methanoculleus* sp. 25XMc2, were also found in *mcrA* clone libraries retrieved from the same DNA samples in this study [24].

Six out of ten isolates and enrichment culture clones of hydrogenotrophic methanogens were obtained from initial slurry cultures supplemented with methylated compounds and subsequent H_2_/CO_2_-amended cultures (Supplementary Table S5). Only one hydrogenotrophic methanogen, strain 12X3c12, was obtained from the initial slurry culture with H_2_/CO_2_. Some acetogenic bacteria are known to oxidize methanol to H_2_/CO_2_ in coculture with hydrogenotrophic microorganisms [46, 47], suggesting that many of the hydrogenotrophic methanogens isolated or detected in this study grew with syntrophic methanol oxidation in the initial cultures supplemented with methylated compounds. Indeed, bacterial clones solely detected in these cultures were closely related to an acetogenic bacterium, *Acetobacterium carbinolicum* [48] (99% sequence similarity in the 16S rRNA gene). Accordingly, methanol might be an indirect methanogenic substrate for hydrogenotrophic methanogens, which are utilized by the bacterium to yield hydrogen. Because the H_2_ partial pressure on syntrophic methanol oxidation was lower than the direct supplementation of H_2_ under the examined conditions, many hydrogenotrophic methanogens would have been unable to grow in the initial culture when suddenly exposed to a high substrate (H_2_) concentration, as previously suggested [49].

Methanogen isolates were also successfully obtained from the slurry samples initially cultivated without the addition of methanogenic substrates, followed by subculturing with the substrates (Supplementary Table S5). The methanogenic isolates and clones assigned to hydrogenotrophic *Methanobacterium* and *Methanococcus* and obligatory acetoclastic *Methanosaeta* originated only from these cultures.

### Methanogenesis from sediment slurries without any supplementation

The sediment slurry sample from 43 mbsf that showed the highest potential methanogenic activity was incubated without the addition of substrates. Incubation at 25 °C for 14 months resulted in methane production up to 4.3 µmol g^–1^ sediment (Supplementary Fig. S1).

The slurry sample after 14 months of incubation was subjected to clone library and quantitative PCR analyses based on archaeal 16S rRNA genes. Only two archaeal sequences were obtained: the sequences of these clones were identical to those of the isolated strain *Methanosarcina* sp. MSS35 (42 out of 46 clones) and *Methanolobus* sp. 17PMc2 (4 clones). Strain MSS35 utilized acetate and methylated compounds, whereas strain 17PMc2 utilized only methylated compounds, suggesting that methane was produced via the methylotrophic pathway or methylotrophic and acetoclastic pathways in the 43 mbsf slurry sample. After incubation, archaeal 16S rRNA gene copy numbers were 2.6 × 10^5^ g^–1^ of sediment, which was two orders of magnitude higher than those in the original 43 mbsf sediment sample [24].

Based on porewater chemistry data [24], at the start of this incubation, the slurry sample is estimated to have contained approximately 0.02 µmol g^–1^ acetate derived from the sediment porewater. The amount of methane that can theoretically be produced from the conversion of this porewater acetate is much lower than that of actually produced methane. Collectively, these results suggest that the degradation of organic matter coupled to methylotrophic (and acetoclastic) methanogenesis occurred in the long-term batch incubation of the 43 mbsf sediment sample without any substrate supplementation.

### Temperature characteristics of methane production in methanogenic isolates

**Fig. 4:**
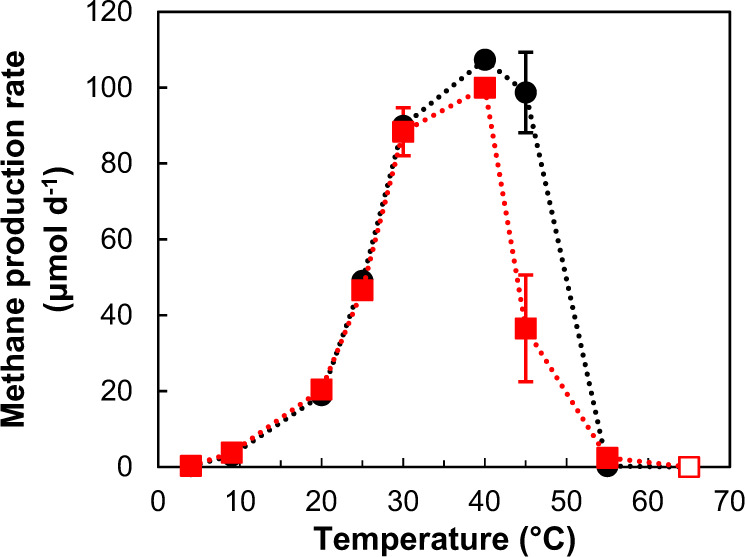
Temperature dependence of the methane production rate in hydrogenotrophic methanogenic isolates. Open symbols indicate no methane production. Symbols: black circle, *Methanocalculus* sp. 1H1Hc7; red square, *Methanoculleus* sp. 25XMc2.

The methanogenic activity levels at different temperatures, corresponding to different sediment depths, were examined using two methanogenic isolates, *Methanocalculus* sp. 1H1Hc7 and *Methanoculleus* sp. 25XMc2. These two hydrogenotrophic strains were selected because of their predominance and prevalence in the original sediment core samples (Supplementary Table S5). Both isolates produced methane at 4–55 °C, with the production rate maximized at 40–45 °C (Fig. 4). Assuming that the geothermal gradient is 0.03 °C m^–1^.

in the eastern Nankai Trough [27], this result indicates that these strains have the ability to produce methane at in situ temperatures and is consistent with the depth profiles of ^14^C tracer activities and F430 concentrations. Similar temperature dependence of the methane production rate has been reported for other hydrogenotrophic methanogens, such as *Methanocullues submarinus* and *Methanococcus aerolicus* isolated from the eastern Nankai Trough sediments, although their relative abundances in natural settings have not yet been assessed [15, 17].

## Discussion

To estimate the diversity and potential activity of methanogenic microorganisms in the methane hydrate-concentrated zone, subseafloor sediment should be collected with caution against contamination from seawater and drilling fluids. This is especially important in this study because contamination control tracers could not be used during coring/sample collection and because the coring methods using extended core barrels are disruptive to the cores. Therefore, we carefully selected core samples to minimize the risk of contamination: X-ray CT scanning was performed immediately after sample collection, and part of the samples showing neither cracks, disturbance, nor traces of drilling fluid was used for further analyses. As a result, all core samples were collected exclusively from mud layers. We further removed the core sample surfaces (approximately 3 cm thick) and only used the sediments in the centre for all microbiological experiments. In addition, we checked the Illumina 16S rRNA gene sequence data to confirm the absence of sequences assigned to the SAR11 clade and the genus *Xanthomonas* indicative of contamination from seawater and drilling fluids, respectively [24]. Indeed, no such sequences were detected in the data from all the samples used.

Amplicon sequencing and long-term batch incubation of 30 sediment samples revealed that methanogens in the subseafloor sediment of biogenic gas hydrate deposits are phylogenetically and metabolically more diverse than previously found. The use of indirect methanogenic substrates, which are utilized by bacteria to yield substrates for methanogens, was more effective in culturing methanogens than the use of direct substrates, especially for hydrogenotrophic methanogens. Imachi et al. [50] reported that an initial enrichment culture of subseafloor sediments using a continuous-flow-type cultivation system supplemented with indirect methanogenic substrates resulted in successful culturing of methanogens, whereas the initial cultures using a batch-type system did not. Our results indicated that initially using a batch-type cultivation system supplemented with indirect methanogenic substrates is also effective in culturing methanogens from subseafloor sediments.

The carbon isotopic ratios of methane and DIC suggest that methane in the studied sediments was of a biogenic origin via the carbonate reduction pathway. Both radiotracer experiments and molecular analyses consistently indicated the predominance of hydrogenotrophic methanogenic pathways in the sediments. The predominance of hydrogenotrophic methanogenic activity based on radiotracer experiments was also found in sediments from the Cascadia margin [5] and the central Nankai Trough [51]. This study revealed that two hydrogenotrophic methanogens, *Methanocalculus* and *Methanoculleus*, that were dominant in methanogenic communities in sediment samples showed their upper limits for methanogenesis at temperatures of approximately 45–50 °C. This trend is in good agreement with radiotracer experiments in previous studies [7, 51]: potential methanogenic activity in sediments at four sites in the central Nankai Trough markedly decreased at depths corresponding to an estimated temperature of approximately 45 °C. The occurrence of in situ methanogenesis at sediment depths corresponding to these temperatures is among the important targets for future study.

The detection of methanol in sediment porewater, the methylotrophic methanogenic activity from methanol and the culturable obligatory methylotrophic methanogens from various sediment depths and from incubated sediment samples without any supplementation all indicate the potential for methylotrophic methanogenesis. Consistent with our results, previous studies have detected evidence for methylotrophic methanogenesis in deeply buried sediments: conversion of radiolabelled methanol to methane in the sediments from the central Nankai Trough [51] and the Japan Sea [21], transcripts for enzymes involved in methylotrophic methanogenesis in sediment from the Peru Margin sediment [52] and *mcrA* gene sequences related to methylotrophic methanogens in sediments off Shimokita Peninsula [14]. Our results further suggest the potential for methylotrophic methanogenesis from methanol in deeply buried sediments. These findings provide insight into subsurface methanogenesis because the occurrence of methylotrophic methanogenesis has focused on the surface [53] but not on deep-buried sediments.

In conclusion, this study reveals the previously overlooked phylogenetic and metabolic diversity of methanogens in subseafloor sediments in biogenic gas hydrate areas. These lines of evidence along with future examinations of the occurrence and in situ activity of methanogens in deeper sediments will provide the vertical profile of methanogenesis, a key issue for understanding marine gas hydrate formation.

## Supplementary information

Supplementary Information
