# Supplementary material for: Cultivation and biogeochemical analyses reveal insights into methanogenesis in deep subseafloor sediment at a biogenic gas hydrate site
Source: ISME J. 2022 Feb 2;16(5):1464–72. doi: 10.1038/s41396-021-01175-7 (PMC9038717; doi:10.1038/s41396-021-01175-7)
Supplement: Supplementary file 1 — Supplementary Information [file 41396_2021_1175_MOESM1_ESM.pdf]

Supplementary Information for

Cultivation and biogeochemical analyses reveal insights into methanogenesis in deep subseafloor sediment at a biogenic gas hydrate site

Taiki Katayama, Hideyoshi Yoshioka, Masanori Kaneko, Miki Amo,
Tetsuya Fujii, Hiroshi A. Takahashi, Satoshi Yoshida and Susumu Sakata

Correspondence to: Yoshioka H, hi-yoshioka@aist.go.jp

This file includes:

Supplementary Figure S1. Time course of methane formation in sediment core slurry sample (43 mbsf) without any supplementation.

Supplementary Table S1. Primers used in this study.

Supplementary Table S2. The stable carbon isotopic ratios of methane and dissolved inorganic carbon in sediment and porewater samples, respectively.

Supplementary Table S3. The methanol concentrations in porewater samples.

Supplementary Table S4. Levels of methane production after the cultivation of the sediment slurry samples with methanogenic substrates.

Supplementary Table S5. Characteristics of the methanogen isolates and culture clones.

Supplementary Figure

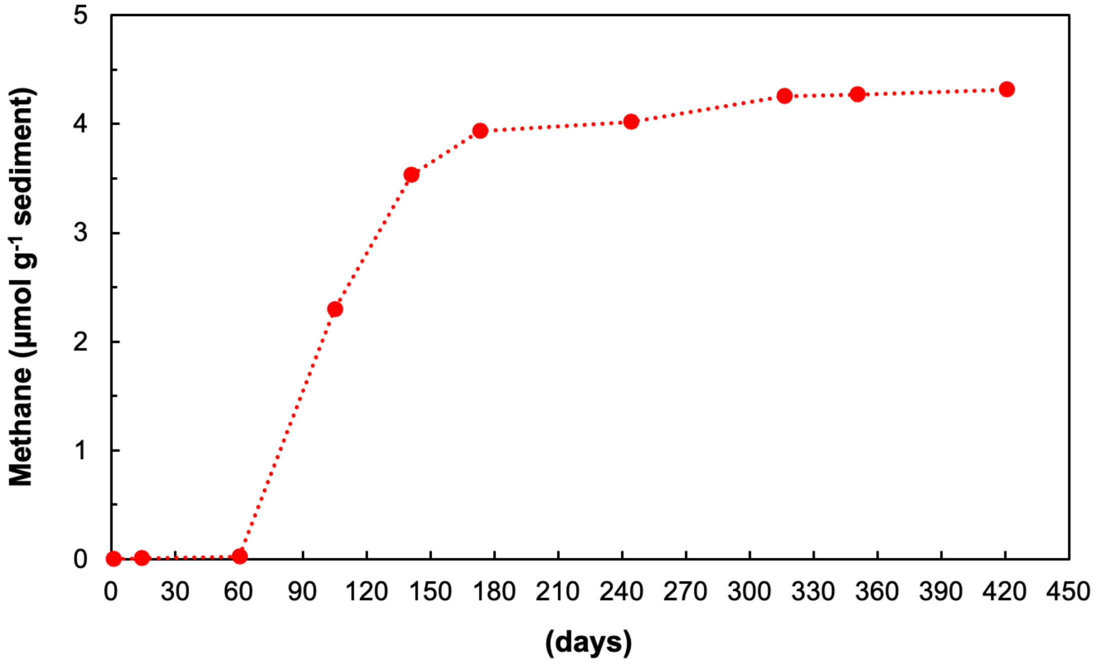

Figure S1. Time course of methane formation in sediment core slurry sample (43 mbsf) without any supplementation.

Supplementary Tables

Table S1. Primers used in this study.

Primer	Sequence (5' to 3')	Target gene
Arc806R	GGACTACVSGGGTATCTAAT	archaeal 16S rRNA gene
Arc109F	AMDGCTCAGTAACACGT	archaeal 16S rRNA gene
Arc21F	TTCCGGTTGATCCYGCCGGA	archaeal 16S rRNA gene
Univ1490R	GGHTACCTTGTTACGACTT	archaeal 16S rRNA gene
Bac8F	AGAGTTTGATCMTGGCTCAG	bacterial 16S rRNA gene
Bac1492R	TACGGYTACCTTGTTACGACTT	bacterial 16S rRNA gene
ME3MF	ATGTCNGGTGGHGTMGGSTTYAC	*mcrA* gene
ME2r’	TCATBGCRTAGTTDGGRTAGT	*mcrA* gene

Table S2. The stable carbon isotopic ratios of methane and dissolved inorganic carbon in sediment and porewater samples, respectively.

Borehole	Depth (mgsf)	$\delta^{13}C-CH_4$ (‰ vs. VPDB)	$\delta^{13}C$-DIC (‰ vs.VPDB)	Difference
AT1-GT	1	-82.4	-45.9	-36.5
	11	-70.4	8.0	-78.4
	43	-66.7	11.0	-77.7
	84	-69.8	10.1	-79.9
	122	-67.7	11.6	-79.3
	179	-69.0	8.3	-77.3
	259	-67.3	9.7	-77.0
AT1-C	282	-68.1	10.1	-78.2
	295	-65.0	9.6	-74.6
	300	-65.1	11.5	-76.6
	307	-61.1	10.4	-71.4

Table S3. Methanol concentrations in porewater samples.

Depth (mbsf)	(uM)
1	6.7
43	6.2
94	9.2
171	8.0
272	75.6

Table S4. Methane production levels after the cultivation of the sediment slurry samples with methanogenic substrates.

Borehole	Depth (mbsf)	% of maximum theoretical yield of methane			
		H2+Ace	H2+Ace +Anti	Meth+TMA	Meth+TMA +Anti
AT1-GT	1	-	-	30	-
	2	0.04	-	52	-
	5	-	-	-	76
	11	-	-	-	-
	16	0.07	-	-	77
	19	-	-	-	-
	30	-	-	-	-
	43	0.07	-	56	-
	65	-	-	-	79
	76	-	-	-	77
	84	-	-	-	-
	94	0.06	-	-	-
	103	-	-	37	-
	116	-	-	-	-
	122	-	-	71	-
	141	-	-	-	-
	171	-	-	-	-
	179	0.10	-	-	-
	198	0.10	-	-	-
	216	-	-	54	-
	241	-	-	-	-
	249	-	-	-	-
	259	-	-	-	-
	272	-	-	-	76
AT1-C	260	-	-	-	-
	273	-	-	-	-
	282	0.03	-	-	-
	295	-	-	-	-
	300	-	-	82	-
	307	0.04	-	-	-

Abbreviations; Ace, Acetate; Anti, Antibiotics; Meth, Methanol; TMA, Trimethylamine

Table S5. Characteristics of the methanogen isolates and culture clones.

Strains and clones	Origins (mbsf)	Closest species (% of sequence similarity)	Culture conditions		Abundance (%) relative to total methanogen sequences in the original sediment samples (mbsf)							
			Initial culture	2nd/Isolation								
			Substrates (°C)	Substrates (°C)	1	11	43	259	282	295	300	307
**Methanogen isolates**												
13XMc1	103	*Methanosarcina semesiae* (98)	Meth+TMA (9)	Meth+TMA (25)	-	-	-	-	-	-	-	-
MSS35	43	*Methanosarcina semesiae* (98)	No add. (25)	Meth+TMA (25)	-	-	0.078	-	1.1	0.52	-	7.7
17PMc2	300	*Methanolobus profundi* (99)	Meth+TMA (9)	Meth+TMA (25)	-	-	-	-	-	-	-	-
Mtav32	43	*Methanosaeta harundinacea* (99)	No add. (25)	Acetate (25)								
6TMc1	43	*Methanoplanus limicola* (100)	Meth+TMA (9)	H_2+CO_2 (25)	-	-	-	-	-	-	-	-
1H2c2	2	*Methanoculleus taiwanensis* (95)	Meth+TMA (9)	H_2+CO_2 (25)	-	-	-	-	-	-	-	-
12X3c12	94	*Methanoculleus taiwanensis* (98)	H_2+CO_2+Acetate (9)	H_2+CO_2 (25)	-	-	-	-	-	-	-	-
25XMc2	216	*Methanoculleus marisnigri* (99)	Meth+TMA (9)	H_2+CO_2 (25)	-	15	2.3	-	-	2.4	-	22
1H1Hc7	1	*Methanocalculus pumilus* (99)	Meth+TMA (9)	H_2+CO_2 (25)	-	-	50	76	53	3.8	0.48	42
Mba6	43	*Methanobacterium movens* (99)	No add. (25)	H_2+CO_2 (25)	-	-	-	-	-	-	-	-
**Enrichment culture clones**												
6T1cc2_A11	43	*Methanosaeta harundinacea* (98)	No add. (25)	Acetate (25)	-	-	-	-	-	-	-	-
2A1_A29	216	*Methanosarcina lacustris* (99)	Meth+TMA (9)	-	-	-	-	-	-	-	-	-
1H1MHA_A01	1, 2, 103, 216	*Methanococcoides alaskense* (99)	Meth+TMA (9)	Meth+TMA (25)	-	-	-	-	-	-	-	-
15XMHA_A06	43	*Methanolobus profundi* (98)	No add. (25)	-	-	-	-	-	-	-	-	-
3H3MHA_A01	5, 16, 65, 76, 216, 272	*Methanolobus profundi* (99)	Meth+TMA+Anti. (9)	Meth+TMA (25)	-	-	-	-	-	-	-	-
1H2MHA_A06	2	*Methanoplanus petrolearius* (99)	Meth+TMA (9)	H_2+CO_2 (25)	-	-	-	-	-	-	-	-
6T1cc2_A21	43	*Methanogenium marinum* (99)	No add. (25)	H_2+CO_2 (25)	-	-	-	-	-	-	-	-
13XMHA_A07	103, 122	*Methanocalculus pumilus* (99)	Meth+TMA (9)	H_2+CO_2 (25)	-	-	-	-	-	-	-	-
6T1cc2_A06	43	*Methanococcus vannielii* (99)	No add. (25)	H_2+CO_2 (25)	-	-	-	-	-	-	-	-

Abbreviation; Anti, Antibiotics.
